# Consumption of Plant-Derived Phenolic Acids Modulates Hunger and Satiety Responses Due to Chemical Interactions with Enteroendocrine Mediators

**DOI:** 10.3390/foods13223640

**Published:** 2024-11-15

**Authors:** B. Shain Zuñiga-Martínez, J. Abraham Domínguez-Avila, Marcelino Montiel-Herrera, Mónica A. Villegas-Ochoa, Rosario Maribel Robles-Sánchez, J. Fernando Ayala-Zavala, Manuel Viuda-Martos, Gustavo A. González-Aguilar

**Affiliations:** 1 Centro de Investigación en Alimentación y Desarrollo A. C., Carretera Gustavo Enrique Astiazarán Rosas No. 46, Col. La Victoria, Hermosillo 83304, SO, Mexico; shain.zuniga21@gmail.com (B.S.Z.-M.); mvillegas@ciad.mx (M.A.V.-O.); jayala@ciad.mx (J.F.A.-Z.); gustavo@ciad.mx (G.A.G.-A.); 2 CONAHCYT-Centro de Investigación en Alimentación y Desarrollo A. C., Carretera Gustavo Enrique Astiazarán Rosas No. 46, Col. La Victoria, Hermosillo 83304, SO, Mexico; 3 Departmento de Medicina y Ciencias de la Salud, Universidad de Sonora, Blvd. Luis Encinas y Rosales s/n, Col Centro, Hermosillo 83000, SO, Mexico; marcelino.montiel@unison.mx; 4 Departmento de Investigación y Posgrado en Alimentos, Universidad de Sonora, Blvd. Luis Encinas y Rosales s/n, Col Centro, Hermosillo 83000, SO, Mexico; maribel.roblessanchez@unison.mx; 5 IPOA Research Group, Instituto de Investigación e Innovación Agroalimentaria y Agroambiental (CIAGRO-UMH), Universidad Miguel Hernández, 03312 Alicante, Spain; mviuda@umh.es

**Keywords:** enteric hormones, feeding, fruits and vegetables, hunger, ultra-processed foods

## Abstract

Energy-dense foods are commonly rich in fat and simple sugars and poor in dietary fiber and micronutrients; regularly consuming them decreases the concentration and/or effect of anorexigenic hormones and may increase that of orexigenic ones, thereby decreasing satiety. In contrast, plant-derived phenolic-rich foods exert positive effects on satiety. In silico, in vitro, and in vivo investigations on some of most representative phenolic acids like chlorogenic acid (CGA), gallic acid (GA), ferulic acid (FA), and protocatechuic acid (PCA) have shown that they are able to modulate various hunger and satiety processes; however, there are few studies that show how their chemical structure contributes to achieve such effects. The objective of this review is to summarize how these phenolic acids can favorably modulate hormones and other satiety mediators, with emphasis on the chemical interactions exerted between the core of these compounds and their biological targets. The evidence suggests that they form interactions with certain hormones, their receptors, and/or enzymes involved in regulating hunger and satiety, which are attributed to their chemical structure (such as the position of hydroxyl groups). Further research is needed to continue understanding these molecular mechanisms of action and to utilize the knowledge in the development of health-promoting foods.

## 1. Introduction

High-energy, low-micronutrient foods, such as processed snacks, sweets, and high-sugar beverages, have been shown to alter the secretion and effect of hormones that regulate hunger and satiety, thereby increasing food intake [1]. These so-called ultra-processed foods have even been shown to significantly alter the enteral and neuronal pathways that regulate an organism’s response to macronutrients, including the reward system, in a way that promotes the further consumption of such foods and beverages [2]. Conversely, forming healthy eating habits can be an aid in controlling hunger and cravings, thereby preventing the overconsumption of unhealthy foods [3].

Food intake is a highly regulated and complex process that is overall mediated by hunger, satiety, and appetite signals from peripheral tissues and integrated by the brain. Hunger is a physiological demand for nutritional replenishment generated by metabolic processing, which in turn stimulates a drive for food intake [4]. Satiety is the sensation of feeling full after the resolution of caloric deficiency [4,5]. Appetite, in turn, refers to a psychological desire to eat and engages diverse cognitive and socio-environmental signals [6]. They are all regulated by homeostatic and non-homeostatic signals that are driven by the brain–intestine–adipose tissue axis [7]. The importance of satiety in regulating food intake and some diet-related diseases, such as obesity, has led researchers to develop tools to promote it, although one of the simplest and most effective is through adequate healthy habits that prioritize plant-based foods. This is a concerning problem that is associated with multiple other detrimental effects, including hypertension, diabetes and others, which have a significant impact on the individual, health systems, and society as a whole.

Foods from vegetable sources are rich in phenolic compounds and, in particular, phenolic acids like chlorogenic acid (CGA), gallic acid (GA), ferulic acid (FA), and protocatechuic acid (PCA), which are chemically simple but highly bioactive molecules. These compounds are present in a wide variety of foods, with their concentrations depending on species, processing, extraction methods, and multiple other variables. For example, CGA is particularly abundant in some green coffee beans, where their concentration is reported to be up to 14.4% dry weight (dw) in *Coffea canephora*, in contrast to notably low values of 0.61% dw in *Coffea* sp. Bakossi [8]. GA can be found in grape juice, raspberries, and strawberries in concentrations of up to 110, 102 and 89 mg/kg, respectively; polymeric structures (hydrolyzable tannins) are also abundant, although these are not considered GA sources for the purposes of the present work. FA is characteristic of cereals and, more specifically, of its bran more than its endosperm, with the former having significantly higher content than the latter. For example, Roma and Almari wheat bran contain 989 and 888 μg/g, respectively, while their flour contains only 183 and 343 μg/g, respectively [9]. Notable concentrations of PCA were reported in *Euterpe oleracea* oil (Acai), with values of up to 630 mg/kg [10]; moreover, cyanidin glycosides may be biotransformed into PCA, thereby making anthocyanin-rich sources (such as different berries) PCA-rich sources as well [11].

Studies have been performed to determine their effects on health, most of which mainly focus on their antioxidant properties, although recent research has revealed additional bioactivities, including anti-inflammatory, hypoglycemic and, of particular relevance to the present work, a potential to induce satiety through different mechanisms [12,13]. These studies showed effects related to inducing satiety and reducing hunger, thereby promoting a voluntary reduction in food intake [14]. Although there is evidence about the mechanisms by which these effects are carried out, there are still significant knowledge gaps; thus, it is important to study them as a strategy for the prevention and treatment of diet-related non-communicable diseases like obesity. The objective of the present work is to analyze some of the physiological bioactivities of four selected phenolic acids regarding their impact on hunger and satiety processes while also summarizing evidence about how their chemical structure contributes to these effects. Some knowledge gaps are also identified in order to guide future studies on this topic.

A list of abbreviations is given at the end of the document.

## 2. Diet-Induced Disturbances on Hunger and Satiety Pathways

There is currently a growing problem regarding the excessive consumption of low-fiber foods that are high in calories, simple sugars, and saturated fats, which, in addition to a sedentary lifestyle, cause a negative effect on satiety that can evolve into serious health problems like obesity [15]. The latter begins with the overconsumption of such foods, which promotes the accumulation of white adipose tissue and further increases food intake, as well as an increase in the production of reactive oxygen species that trigger oxidative stress [16]. These factors then contribute to a decrease in the antioxidant defense system, inflammation, and altered response to chemical mediators of hunger and satiety, finally converging on the onset and progression of various non-communicable diseases [17].

Such foods reduce the secretion, circulating concentration, and/or effect of satiety-inducing hormones like glucagon-like peptide-1 (GLP-1), peptide tyrosine-tyrosine (PYY), and cholecystokinin (CCK) from enteroendocrine cells of the intestine; the effect of adipose-derived leptin and its receptor (LEPR) is also altered, resulting in decreased anorexigenic signaling. These hormones have a systemic effect on the organism’s energy metabolism and multiple other physiological processes [18]. Some negative effects are also apparent on certain areas of the brain that integrate peripheral hunger and satiety signaling, for instance, hypothalamic receptors of anorexigenic signals in the arcuate nucleus (ARC) can be downregulated by the consumption of high-fat/low-fiber foods, resulting in an unrestrained effect of orexigenic signals (e.g., ghrelin and cortisol) [19]. Therefore, chronic exposure to a high-fat, low-fiber diet decreases and disturbs satiety modulation, according to altered central and peripheral signaling as previously described. This phenomenon makes it highly important to comprehend the mechanisms underlying satiety regulation where, following food intake, diverse signals work to enhance sensory thresholds and impact neural circuits governing this process, in order to maintain health.

## 3. Impact of Phenolic Compounds on Satiety Regulation

In contrast to consuming unhealthy foods, favoring a diet rich in phenolic compounds has been shown to induce multiple benefits associated with the aforementioned peripheral and central pathways, in addition to various other health effects [20]. These molecules are functional components that are widely found in plant foods, including fruits, vegetables, and whole grains, among others. Evidence from in vitro and in vivo studies has contributed to elucidate the potential role of some phenolic compounds on gut–brain axis hormones that modulate food intake and energy metabolism [21]. For example, cell and animal models have shown that (-)-epicatechin and anthocyanins promote GLP-1 activity by stimulating its mRNA expression and decreasing dipeptidyl peptidase 4 (DPP4) mRNA expression (the main inactivating enzyme of GLP-1), altogether improving GLP-1 signaling [22]. Phenolic compounds can also function as ligands of G-protein-coupled receptors (GPCRs), such as FA, which can interact with specific ghrelin receptor residues, as well as epigallocatechin gallate, which interacts with specific GLP-1 receptor residues [23,24]; the activation of these receptors can delay gastric emptying and slow down gastrointestinal motility, ultimately inducing satiety [23].

**Figure 1:**
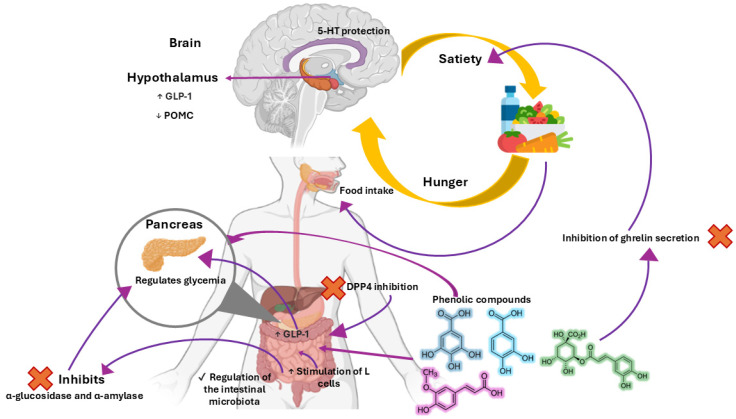
Overall mechanisms of satiety regulation exerted by phenolic acids considered in the present work. Hunger signals originate partly from the stomach, which are regulated through vagus signaling. When consuming food, phenolic compounds are released in the gastrointestinal tract, where they begin to exert their effects, for example, by decreasing the secretion of ghrelin to inhibit hunger. They can also stimulate L cells in the distal small intestine, therefore increasing the concentration of GLP-1, as well as inhibiting the enzyme DPP4 once in circulation. They inhibit the enzymatic activity of intestinal α-glucosidase and α-amylase, which, along with GLP-1’s insulin-stimulating effects, can modulate carbohydrate metabolism. They positively regulate the intestinal microbiota, whose metabolites can also induce satiety. In the hypothalamus, they can protect 5-HT, increase GLP-1 mRNA, and decrease POMC mRNA.

Evidence from animal experiments has shown that different phenolic compounds are able to induce anti-obesity effects, in part by leading to a voluntary reduction in food intake, subsequently reducing their body weight and/or adiposity, but the exact mechanisms behind these effects require further study. For example, Serna et al. [25] reported that some vegetable extracts rich in phenolic compounds have appetite-suppressing effects, which have been attributed to different converging mechanisms like slowing down the secretion of orexigenic hormones and increasing that of anorexigenic ones, in addition to inactivating appetite sensors, all of which converge on relaying satiety signals to the brain [26]. The following sections describe reported mechanisms of four selected phenolic acids on hunger and satiety and is graphically summarized in Figure 1.

## 4. Bibliographic Analysis

The Scopus and Web of Science databases were consulted in order to include only high-quality peer-reviewed papers. We searched for “chlorogenic acid”, “gallic acid”, “ferulic acid”, and “protocatechuic acid”, in addition to “hunger”, “satiety”, “hormones”, “insulin”, “cholecystokinin”, “CCK”, “GLP-1”, “GLP1”, “peptide tyrosine-tyrosine”, “PYY”, “dipeptidyl peptidase 4”, “DPP4”, “receptor”, “digestive enzymes”, “digestion”, “postprandial”, “receptor”, “in silico”, “interaction”, and “docking”. Resulting papers were then carefully analyzed in order to determine if the analyses reported conformed to the topic of the present work.

## 5. Reported Effects of Four Selected Phenolic Acids on Hunger and Satiety

### 5.1. Chlorogenic Acid (CGA)

CGA is a phenolic acid present in plant-based foods and beverages, including coffee, green tea (*Camellia sinensis*), and yerba mate (*Ilex paraguariensis*), among others. CGA was reported to have antioxidant, antihypertensive, lipid-lowering, anti-inflammatory, and weight-regulating properties [27,28].

There is evidence that CGA-rich sources exert satiety effects in humans. For example, Celestino et al. [29] conducted a single-blind randomized study, where 10 overweight and obese women were administered three tablets of yerba mate, guarana (*Paullinia cupana*), and damiana (*Turnera diffusa*) extract. They found that, two days after the extracts were consumed, GLP-1 concentrations were higher than the control group, after 60 and 150 min of consuming a standardized meal. GLP-1 stimulates pyloric contraction, resulting in delayed gastric emptying and reduced gastrointestinal transit, in addition to stimulating the secretion of amylin, which also reduces gastric emptying by vagal input [30], thereby promoting satiety-inducing effects. Such evidence is relevant due to the fact that a significant impact on humans is reported; however, effects on 10 participants may not be sufficient to generalize these results, which suggests the need for additional evidence. Another observation that can be made is that the documented effects are most likely due to other compounds in addition to CGA, either by themselves or through an additive or synergistic mechanism, since the treatment was not administered in a pure form, although administering a plant extract approximates the way in which people normally consume them (for example, as teas or infusions).

A similar study by Yanagimoto et al. [31] reports the effects of green tea CGA and catechins on 11 healthy men. After consuming a beverage with 373 mg of CGA and 620 mg of catechins (in addition to 119 mg of caffeine) for three weeks, the authors report an improved insulin sensitivity, according to a maximal postprandial glycemia (C_max_) of 105.5 ± 4.6 mg/dL in response to the treatment, in contrast to 118.5 ± 5.2 mg/dL for the placebo group. Increased fasting GLP-1 concentration was also documented, according to an area under the curve (AUC) of 37.2 ± 4.2 pmol/L·4 h for the treated group, as compared to 25.3 ± 3.2 pmol/L·4 h for the placebo group. The significant changes to this hormone and its effects are promising, although the effects on satiety were not specifically analyzed and therefore cannot be confirmed or denied with these data. Similar to a study conducted by Celestino et al. [29], these findings are relevant due to the way in which the treatment was administered to human participants and at a dose that can be realistically obtained from food, although the sample size was also limited.

Evidence from animal models also suggests that CGA can stimulate GLP-1 under certain conditions; for example, Sharma et al. [32] administered 50 mg/kg of CGA to mice with rotenone-induced Parkinson’s disease for 13 weeks. After the experimental period, the authors report an increase in circulating GLP-1, but only when rotenone and CGA were co-administered; although neither compound was able to significantly increase GLP-1 concentration by itself, there is a clear tendency that suggests a slight effect by CGA, but not by rotenone. Interestingly, the effects of GLP-1 were associated with improvements in Parkinson’s-related parameters, while its actions on satiety were not specifically analyzed.

In vitro and in silico data complement the aforementioned animal- and human-derived findings and can help explain the effects observed. Grzelczyk et al. [33] analyzed the inhibitory effects of CGA derivatives found in coffee (including esters of caffeic acid and FA), on the enzymatic activity of monoamine oxidase A (MAO-A). This enzyme is responsible for the deamination of biogenic amine neurotransmitters like 5-hydroxytryptamine (serotonin, 5-HT) in the central nervous system. The authors highlight how alterations in 5-HT have been associated with satiety disorders and, more specifically, with the increased consumption of simple sugars that can promote obesity, diabetes, and other related diseases. The authors isolated coffee bioactives, performed isometric titration calorimetry and molecular coupling analyses, and determined that they are optimal candidates to inhibit MAO-A in vivo and protect 5-HT from deamination. The results suggest the potential of coffee bioactives to act on the central nervous system, but its processing can significantly alter it, since green coffee was the most suitable one to potentially decrease sugar consumption [33,34]. This evidence is consistent with previous observations that link coffee consumption with a decreased risk of diabetes, and, although caffeine has been considered as a potential contributor to this effect, CGA is also known to have a favorable impact on carbohydrate metabolism by acting on the incretin system (GLP-1) [35].

The effects of CGA on GLP-1 and carbohydrate metabolism, in general, may be due to at least two mechanisms; first, animal studies showed that CGA can increase sodium-glucose cotransporter-1 (SGLT-1) levels, which in turn maintains an adequate glucose homeostasis and is also associated with increased GLP-1 secretion [36]. Second, a suppressive effect of CGA was proposed on glucose absorption in the small intestine, which could shift the site of absorption to the distal small intestine, increasing L cell stimulation and, therefore, a greater GLP-1 secretion [32]. It should also be stated that, due to the vegetable sources where it is abundant (such as coffee and tea), its effects may also be associated with those of caffeine and/or other phenolics, including the ones discussed in the present work, which can exert an additive or synergistic effect.

### 5.2. Gallic Acid (GA)

GA is a phenolic acid commonly found in tea leaves and in various fruits like grapes and berries; it has shown antibacterial, hepatoprotective, antihyperglycemic, and cardioprotective properties, among others [37].

Evidence where the effect on human hunger and satiety can be specifically attributed to GA is lacking; although it is known that fruits and vegetables, in general, may decrease hunger and promote satiety [14,38], the specific contributions of GA have yet to be conclusively determined in humans. GA and other phenolics have been studied for their potential antidiabetic effects [23,39], some of which are due to modulating GLP-1 (and others), but unequivocally establishing its role on human satiety and the associated molecular targets or pathways is currently a research area that merits further investigation. In contrast, there is robust evidence from animal models that shows how GA can act on different satiety mediators through multiple mechanisms. For example, Serrano et al. [40] reported that a GA-rich grape seed extract sub-chronically (1 g/kg dose for 8 days) reduced food intake in Wistar rats. They found that the compounds in the extract could affect the serum concentration of hunger- and satiety-regulating hormones. The authors specifically mentioned that GA and oligomeric flavanols inhibit ghrelin secretion and could play a major role in long-term in vivo octanoyl ghrelin inhibition exerted by the grape seed extract. In addition to in vivo experiments, they also analyzed the ex vivo (duodenum and colon) and in vitro (MGC3-1 cells) effects of individual compounds, including GA, and found that 10 μM of this molecule was able to decrease the release of octanoyl ghrelin. These results suggest that GA may be central to the observed effects in the different models analyzed. The different results of this study show remarkable consistency between different models, which makes the evidence more robust regarding the potential of GA to induce satiety. Further experiments suggest that one potential mechanism of action may be due to the compound’s bitter taste, according to the potential of GA (and other phenolics) to act as agonists of the human bitter taste receptors hTAS2R14 and hTAS2R39 [41]; modulating satiety-related hormones through said receptors is currently an active research area that seeks to bridge how organoleptic properties (taste) can alter physiologic (enteroendocrine signaling) and behavioral (eating) processes [40,42].

Sousa et al. [43] reported that GA (100 mg/kg body weight) administered alongside a high-fat diet (60.98% calories from fat, 5.28 kcal/g) decreased body weight in mice, in addition to other effects related to mitigating the negative effects of such a diet. Interestingly, the change in body weight took place even though the animals’ energy intake remained unaffected in response to consuming GA; moreover, their adiposity also decreased in animals fed both normal and high-fat diets. These findings suggest that their lipid metabolism and overall energy balance were favorably altered independently of satiety, although it is possible that an effect could have occurred on some enteroendocrine or central satiety mediators, since these were not specifically analyzed.

Certain non-communicable diseases like diabetes are inherently associated with disturbances in various hunger and satiety hormones, making them relevant models in which to study the effects of phenolics. Bashar et al. [44] reported the beneficial effects of GA on diabetic rats, by administering 20 mg/kg/day for four weeks. They found that GA increased insulin sensitivity, which was apparently associated with decreased serum fetuin-A, according to its negative correlation with GLP-1. Glucose uptake was also improved in insulin-dependent organs, such as the liver, through an increased expression of GLP-1 secretory cells in the terminal ileum, while an increased expression of GLUT4 (and others like Wnt1 and β-catenin) mRNA was also detected in hepatocytes. Although satiety was not specifically analyzed in this study, the effects of GA on GLP-1 and on carbohydrate metabolism are relevant, mainly due to the fact that disturbed satiety is commonly present in diabetic patients. A study conducted by Cázares-Camacho et al. [45] reports the effects of mango pulp (100 g/kg of diet) and mango peel (50 g/kg of diet) as rich sources of GA on diabetic rats, where satiety was specifically addressed. They administered the supplemented diet to rats before or after diabetes was induced and found that either treatment was able to counter diabetic hyperphagia. Specific endocrine mediators to which this change could be attributed were unfortunately not analyzed; notwithstanding, it is remarkable that diabetic hyperphagia was mitigated, since the metabolic derangements (including hunger and satiety responses) of a diabetic organism may be severe. Mango peel and pulp are both rich sources of GA; in fact, this compound was shown to be one of the most bioactive ones in this fruit [46,47]. However, it should be pointed out that mangiferin and other phenolics can also be found in addition to GA, which suggests that the aforementioned effects may not be due only to GA. Regardless, these studies provide evidence about the satiety-inducing effect of GA and some of vegetable sources where it can be naturally found, when analyzed in animal models of diabetes.

It is known that various phenolics can modulate appetite signals by inhibiting enzymes like α-glucosidase and α-amylase, as well as intestinal transporters like SGLT and glucose transporter 2 (GLUT2); these inhibitory effects delay gastric emptying and, hence, can promote satiety [48]. These may be some of the mechanisms by which GA may contribute to the aforementioned satiety-related effects; however, additional research is still required.

### 5.3. Ferulic Acid (FA)

FA is a phenolic acid widely known for it is therapeutic use, according to its reported antioxidant, anti-inflammatory, anti-aging, and neuroprotective effects, among others [49]. It is found in numerous plant sources, such as fruits, vegetables, seeds, and leaves, including wheat and mango, and both the edible parts and byproducts like mango leaves and seed [50].

Similar to GA, human-derived evidence where changes in satiety can be directly attributed to FA is lacking. Other authors have explored the role of FA-rich foods as potential satiety inductors; for example, Zhang et al. [51] described the role of cereals as sources of feruloylated arabinoxylans which, once metabolized in the gut, release significant amounts of free FA. Similarly, Barrea et al. [52] argued that hop-derived bitter compounds (which include FA) may modulate satiety in humans, possibly by acting on GLP-1. This evidence is relevant; however, it may not be sufficient to support the role of FA on satiety in humans, since the effect of FA from fiber cannot be distinguished from that exerted by fiber itself; hop-derived bitter compounds are a complex mixture of numerous phenolics, which once again makes it difficult to ascertain how much of the satiety effect can be attributed to FA and not to other compounds or their interactions. Additional evidence in humans is therefore required to quantify the effect of FA on satiety and its different mediators.

Most of the well-established evidence comes from animal and in vitro models, where the data are more robust. For example, Halter et al. [53] evaluated the effects of FA in birds. They observed a decrease in food intake, which may have been due to changes in a variety of anorexigenic and orexigenic hypothalamic mRNAs like proopiomelanocortin (POMC), galanin, ghrelin, and melanocortin receptor 3 (MC3R); likewise, a specific increase in c-Fos mRNA and a decrease in POMC mRNA were also reported in the ARC. The authors found an increased number of c-Fos-expressing cells, an indicator of neuronal activity within the ARC, suggesting that FA treatment leads to ARC stimulation [54]. The ARC contains orexigenic neurons, such as those that express neuropeptide Y (NPY) and agouti-related peptide (AgRP), and anorexigenic neurons, such as those that express POMC and cocaine and amphetamine-regulated transcript (CART), which primarily integrate a variety of peripheral and central signals to modulate energy intake. An activation of the ARC could therefore imply that a variety of signaling pathways were induced in response to FA, altogether leading to suppressed appetite [53].

Tian et al. [55] observed that a 100 mg/kg of body weight dose of FA by oral gavage to mice fed a high-fat diet suppressed weight gain, significantly increased short-chain fatty acid-producing intestinal bacteria, as well as the mRNA expression of free fatty acid receptor 2 and 3 (FFAR2 and FFAR3). No significant changes were recorded on food consumption; however, the effects observed suggest improvements in associated pathways. The authors propose that the aforementioned effects decreased the bacterial genera related to obesity and serum endotoxin, thereby positively modulating the intestinal microbiota. Maintaining a healthy microbiota can exert favorable effects on satiety, since some microorganisms produce short-chain fatty acids that were reported to induce GLP-1, GLP-2, and PYY. Short-chain fatty acids were also reported to activate the expression of GPCRs on intestinal L cells, which leads to increased secretion of GLP-1 and other enteroendocrine peptides that exert an appetite-regulating effect [56]. Specific compounds like lactate and acetate produced by Lactobacilli and Bifidobacteria can be converted into butyrate, which induces the release of GLP-1 from intestinal L cells while also countering gastrointestinal inflammation by suppressing the nuclear factor kappa-light-chain-enhancer of activated B cell (NF-κB) signaling and other health effects. Other studies also reported that short-chain fatty acid-producing bacteria may enhance GLP-1 secretion by regulating FFAR activities [57]. Thus, FA serves as the substrate from which some bacteria can produce compounds that promote satiety, modulate glucose homeostasis, and decrease inflammation [58].

In addition to promoting the release of GLP-1, it is also possible to increase its half-life and potential bioactivity by inhibiting DPP4, the main GLP-1-inactivating enzyme. DPP4 is expressed in the gastrointestinal tract, kidneys, and endothelial layer of blood vessels; in addition to acting on GLP-1, it can also rapidly inactivate the gastric inhibitory polypeptide (GIP) of the incretin system [59]. Al-Ghamdi and Moselhy [60] reported that intraperitoneal doses of FA (10, 25, or 50 mg/kg of body weight) had an inhibitory effect on DPP4 in diabetic rats, according to improvements in GLP-1-mediated insulin secretion and sensitivity, as well as an inhibition of hepatic gluconeogenesis, glucose-6-phosphate dehydrogenase, and phosphoenolpyruvate carboxykinase 1 (Pck1). These have been reported to be key rate-limiting enzymes of hepatic gluconeogenesis, whose expression is enhanced when there are hunger signals, thereby playing a crucial role in increasing glycemia; thus, their inhibition may regulate hunger and satiety processes. Although it is possible that the documented inhibition of DPP4 may have increased GLP-1 concentration or induced satiety, this possibility was unfortunately not specifically analyzed in this study. Others like An et al. [61] provide evidence that shows that treating mice with FA (30 or 120 mg/kg of body weight) leads to an increased serum concentration of GLP-1; complementary in vitro experiments show that this increase is due to inhibiting DPP4, while direct FA-GLP-1 interactions could have also contributed to the observed effect, although the authors did not analyze how satiety was affected in response to their treatments.

Corella-Salazar et al. [62] evaluated the effects of sub-chronic consumption (eight-weeks) of an avocado paste (AP) extract on the diet of healthy Wistar rats (1 g/kg of diet), which contained a combination of phenolic acids, mainly FA, in addition to PCA and p-coumaric acid. The authors observed significantly increased satiety behavior, according to less food consumption in response to the treatment. Moreover, it is interesting to note that satiety was induced when the treatment was administered as part of either a regular or a high-fat diet. The authors suggest that the satiety effect may have been mediated by an increased plasma concentration and/or the mRNA expression of GLP-1, leptin, and adiponectin. FA and the other compounds may have thus promoted the release of said hormones, since they play a major role in modulating satiety [39]; the fact that changes at the mRNA and protein levels were documented may indicate that the treatment acted by modulating the gene expression of said hormones, as well as the secretion and/or half-life of the proteins.

Foods that contain FA and have shown in vitro DPP4 inhibition, such as sprouted quinoa yogurt [63] and Malabar plum (*Syzygium cumini*) [64], whose consumption resulted in stimulating GLP-1. This evidence suggests that FA-containing foods may indirectly promote satiety by inhibiting the inactivation of GLP-1; however, the role of this specific compound cannot be precisely determined in such a complex matrix where multiple phenolics and other types of compounds are present. It is also possible that FA is able to inhibit some carbohydrate-digesting enzymes, since there is evidence that it exerts a mixed inhibition against α-amylase and non-competitive inhibition against α-glucosidase [65]. Such inhibitory effects could slow down starch digestion and have an impact on postprandial glycemia and, therefore, on appetite regulation. This idea is congruent with reports that show how FA can favorably improve glycemia in animal models [66,67], although the simultaneous influence of FA on glycemia and satiety requires further study.

### 5.4. Protocatechuic Acid (PCA)

PCA has extensive antioxidant, anti-inflammatory, neuroprotective, antitumor, antibacterial, and antidiabetic effects, among others [68].

Ex vivo evidence in the human colon (samples derived from patients who underwent a colectomy) of the effect of a grape seed proanthocyanidin extract was reported by Grau-Bové et al. [69]. The authors treated the samples with 100 mg/L of the extract after they were collected in the operating room and showed that PYY increased from both the ascending and descending colon, while no changes were recorded on GLP-1. The authors also performed similar experiments in pig colons and found that their treatment increased the secretion of PYY, CCK, and GLP-1, when administered at 50 and 100 mg/L (a lower 10 mg/L dose did not exert any effects). The extract contained a mixture of different compounds; thus, it is not possible to determine the exact contribution of any single molecule present therein. To better substantiate their findings, they performed similar experiments with pure compounds. Interestingly, 100 nM of PCA showed a tendency (*p* < 0.1) to decrease GLP-1, although no changes were recorded at lower (10 nM) or higher (1 μM) doses. This evidence supports the role of complex phenolic-rich mixtures (as normally consumed by humans) on the secretion of enteroendocrine mediators of hunger and satiety. Said findings show similarity between animal and human tissue, albeit, when performed ex vivo, making additional findings from humans necessary.

Grzelak-Blaszczyk et al. [70] used industrially derived yellow onion waste as a source of PCA and used it to treat high-fat diet-fed rats (0.15%, four weeks). The authors report various effects, including an improved serum lipid profile and antioxidant-related parameters. Changes in weight were not documented, which may have been due to the short duration of the experiment, although lean mass did decrease in response to the high-fat diet. No changes in diet intake were found; thus, the metabolic improvements found were apparently not related to increasing satiety but may have instead been due to changes in microbiota-produced enzymes. As previously stated, an organism’s microbiota has profound potential to alter its metabolism, including that related to energy balance, making this a potential mechanism of action for the observed effects.

Xiang et al. [71] evaluated the effects of PCA supplementation on high-fat diet-induced obese and insulin-resistant mice. They found various improvements like a reduction in body weight, adipose tissue, and hepatic lipids. No satiety-related effects were documented, according to similar amounts of diet consumed; regardless, carbohydrate and lipid metabolism were positively affected. For example, mitochondrial improvements related to glucose use were reported, in addition to increased fatty acid oxidation in muscle. The evidence for the health-promoting effects of PCA are interesting; however, they were apparently unrelated to altered feeding behavior. Although it is possible that some enteroendocrine mediators like CCK, GLP-1, or others could have exerted some of the observed effects, this possibility was not specifically analyzed. Insulin in particular also contributes to short-term satiety; thus, the increased sensitivity to this hormone could have exerted some effects on hunger and satiety pathways, although this was not specifically studied. One important drawback that the authors highlight about their study is that the dose administered (2.7 mM in the animals’ drinking water) is not achievable in humans through food alone, making supplementation necessary.

Additional effects of PCA were reported when isolated from *Hibiscus sabdariffa* and administered to rats (aqueous *H. sabdariffa* extract, 100, 200, or 400 mg/kg), as reported by Alegbe et al. [72]. In vitro, α-amylase and α-glucosidase showed significant inhibition, which could have contributed to the observed in vivo antidiabetic effects. In this model, protective effects on the pancreas were observed, which could have contributed to the antidiabetic actions of the treatment. GLP-1 promotes pancreatic insulin synthesis and secretion, the inhibition of glucagon secretion, as well as increasing the number of pancreatic β cells. GLP-1-insulin signaling can therefore exert an anorexigenic effect that can also be associated with weight loss [73], since GLP-1 is also known to have effects on the central nervous system that can lead to decreased glycemia by promoting satiety and delaying gastric emptying [74]. Unfortunately, the role of GLP-1 or central effects were not specifically studied.

Al Shukor et al. [75] analyzed interactions between eight phenolics, including PCA and GA, with a CCK type 1 receptor (CCK1R). Chinese hamster ovary (CHO) cells overexpressing the receptor were treated with either sulfated CCK (as positive control) or different phenolics; a CCK1R-mediated calcium flux was used as an indicator of activation. Results showed that no compound tested was able to activate the receptor; in fact, GA (but not PCA) exerted an antagonistic effect. This evidence therefore suggests that directly activating CCK1R may not be one of the pathways by which PCA or GA are able to induce satiety. The role of other hormones and/or their receptors was not analyzed.

## 6. Chemical Properties of Phenolic Compounds Associated with Their Effects on Satiety

Various phenolics may produce satiety effects through physicochemical interactions with different targets, including some enzymes, by forming hydrogen bonds between the hydroxyl groups of the B ring of the phenolic and the enzymatic catalytic residues, or through the formation of π-π interactions [76]. Phenolics can also interact with starch, resulting in macromolecular complexes formed through non-covalent interactions, that could slow down its digestion along the gastrointestinal tract. Oliveira et al. [77] reported that phenolic compounds can bind to the active site of DPP4, which is an effective way of prolonging the circulating half-life of GLP-1 and delay gastric emptying and produce satiety. Furthermore, gliptins (like saxagliptin and various others) are antidiabetic pharmaceutical agents that can also promote satiety, with a mechanism of action based on their effect as competitive inhibitors of DPP4 [78], similar to the effects of phenolics. This section describes possible associations by which the chemical structures of phenolic compounds can confer them the ability to act as satiety modulators; the data are also summarized in Table 1.

Tuersuntuoheti et al. [79] reported the inhibitory effect of CGA on the enzymatic activity of DPP4, which the authors attribute to its two hydroxyl groups on the A ring, as well as possible glycosylation in the quinic acid moiety, since its in silico binding affinity with DPP4 was −8.6 kcal/mol higher than other phenolic compounds of similar chemical composition. The formation of glycosides increases the number of hydroxyl groups, which increases a compound’s hydrophilicity and allows it to form more hydrogen bonds. It was shown that various activities of phenolic compounds are dependent on their hydroxyl groups, as well as the relative location of their chemical moieties around them and the position of their rings [80]. The -COOH, -CH_2_=CH-COOH, -CH_2_-CH_2_-COOH, and -OCH_3_ moieties also affect the activity of phenolic compounds, especially -OCH_3_, since they can form internal hydrogen bonds with hydroxyl groups on the benzene ring, which may affect their interaction with DPP4 and, thus, decrease their inhibitory activity against this enzyme [81]. In the case of CGA, it does not contain -OCH_3_ moieties, which gives it a greater inhibition potential against DPP4, as compared to other similar phenolic compounds. The authors also performed an in silico analysis, which showed that CGA formed nine hydrogen bonds with five amino acids (Arg669, Val207, Glu205, Tyr631, and Tyr54), ten hydrophobic interactions with ten amino acids (Arg358, Ser209, Glu206, Tyr662, Arg125, Asn710, His740, Val656, Tyr666, and Phe357), and a hydrogen bond and hydrophobic interaction with one amino acid (Ser 630), within active pocket residues of DPP4. These results suggest that CGA can interact through hydrogen bonds and hydrophobic interactions with DPP4, which can result in its inhibition, thereby increasing GLP1 circulating half-life and its associated satiety effects.

In another study, Sharma et al. [32] performed an in silico analysis to determine the potential binding of CGA and other phenolics to free fatty acid receptor 1 (also known as GPR-40) and free fatty acid receptor-2 (also known as GPR-43), which were reported to regulate GLP-1 secretion. The binding energy with GPR-40 and GPR-43 showed that CGA had greater affinity, according to −7.38 and −7.47 kcal/mol, respectively, as compared to other phenolic compounds. It was also found that hydrogen bonds with Gln1105 and Glu1022 of GPR-40 broke, while new ones formed with Met1106 and Ser1136, in addition to hydrophobic interactions. Regarding GPR-43, hydrogen bonds with Lys6, Lys65, Glu68, Cys164, and Glu166 were broken, and new bonds were formed with Ser86, Tyr90, and Ser297. It was observed that the interactions with Tyr165 reflect the potential of CGA to increase the activity of GPR-43. According to this observation, CGA appears to have an ideal structure to hydrogen bond to these receptors, allowing it to improve GLP-1 secretion.

Leptin is an anorexigenic hormone that regulates food intake and body weight; its receptor is crucially involved in body weight regulation and obesity. Goudar et al. [82] studied the in silico interactions of phenolic compounds with LEPR and found precise coupling and favorable binding energy for CGA (−41.24 kcal/mol), FA (−33.79 kcal/mol), and GA (−29.92 kcal/mol). Specifically, CGA formed three hydrogen bonds with Arg468, while FA and GA formed hydrogen bonds with Asp617 and Asp475. Regarding CGA, it formed hydrogen bonds with Tyr and Glu, where the hydrogens of the hydroxyl groups of CGA were attracted by the oxygens of these residues. The His residue also formed π-π interactions with the aromatic rings of both structures. FA interacted with Asp617 and Ser470 through hydrogen bonds, where the oxygens of the carboxyl and hydroxyl groups of said residues attracted the hydrogens of FA. FA also formed a π-π interaction with Phe504, and a cation-π interaction with Arg468, due to attractions between Arg and the negative charge of the FA aromatic ring. GA interacted with Ser474, Asp475, and His467 by the means of hydrogen bonds; for example, Ser474 interacts with the hydrogen of the carboxylic group of GA. According to these results, the interaction between phenolic compounds with the LEPR was formed through hydrogen bonds, π-π, and cation-π interactions.

A diet high in simple carbohydrates is associated with obesity and other metabolic diseases; it was reported that phenolic compounds interact with macronutrients through hydrogen bonds and hydrophobic, electrostatic, or ionic interactions, which, in addition to the inhibition of key enzymes, regulate their digestibility. These interactions promote their aforementioned satiety effect while also modulating overall energy metabolism and exerting some antidiabetic actions. The ability of phenolic compounds to exert them depends on their structure, including properties like the number of rings and functional groups, mainly hydroxyl moieties, and their spatial distribution [83]. Adefegha et al. [84] reported the in vitro inhibitory effect of GA and PCA on α-amylase and α-glucosidase, where GA exhibited stronger activity that was attributed to the additional hydroxyl group in a key position in its structure, as compared to PCA. In another study, Alegbe et al. [72] found that the in silico binding energy between GA and α-amylase was −6.20 kcal/mol and −5.80 kcal/mol between PCA and α-amylase. The stability of GA is suggested to be due to hydrophobic interactions with Trp406 at the active site, while that of PCA occurred through hydrogen bonds with Asp327 and His600. These findings further support the previously mentioned importance of the number and location of functional groups, where the additional hydroxyl group of GA gives it a greater capacity to bind to the enzyme. Likewise, [65] performed a molecular coupling between FA and α-amylase and α-glucosidase and reported a binding energy of −5.30 kcal/mol and −5.70 kcal/mol, respectively. They showed that FA bound to the active site of α-amylase, where its carboxyl groups on C3 and C4 formed hydrogen bonds with Gly334, as well as additional hydrophobic interactions. As for its interaction with α-glucosidase, three hydrogen bonds were formed with Asp215, Glu277, and Tyr158, and three π-π interactions with Phe158, Phe178, and Tyr158. The importance of hydroxyl groups when interacting with various enzymes has been reported, and it suggests that phenolic compounds can interact through non-covalent interactions like hydrogen bonds, cation-π interactions, or electrostatic forces [85].

Phenolics have been shown to form interactions with numerous targets; however, the relationship between the compound’s structure and its ability to form interactions that contribute to their potential to regulate hunger and satiety has only been recently considered. This is a field of study that merits further investigation, since it may provide evidence to better understand how our organism responds to dietary components, which may then allow us to promote and maintain health.

## 7. Impact of Model and Phenolic Metabolism

According to the evidence discussed in the present work, the different models used will yield specific information and will have particular advantages and disadvantages for the purposes of analyzing hunger and satiety in response to consuming a particular molecule. In vitro models are among the simplest ones and allow the researcher to fine-tune variables like dose and time; they are particularly useful in determining how stimulating a specific cell type with a compound will alter the cell’s gene expression, as well as protein translation, secretion, or activity. A dose–response analysis can be easy to perform as well as administering combinations of compounds to determine how they interact with each other and how the observed response varies. Multiple repetitions can also be performed, due to the minimal space and reagent requirements. The simplicity of this model may also be a disadvantage, since factors like metabolism and hormonal regulation are not present. Thus, determining if the production, secretion, and/or activity of a compound (such as a hormone) changes in response to a phenolic stimulus does not guarantee that this will occur in a human, where multiple other variables may interact to yield a response.

Animal models are closer to the physiological intricacy of a human, where the effects of other cells and mediators are present, and complex results like hunger and satiety can be measured. Even with this increased similarity, no animal model can precisely mimic a human since even minor differences can have a significant impact on the experimental outcome. Moreover, it is not possible to study as many variables as can be analyzed in vitro, due to the impracticality or sometimes impossibility of having as many experimental groups as with cell lines.

Intervention and even epidemiological studies on humans yield some of the most relevant data. Studies conducted on humans in a laboratory setting can give the researcher increased control over environmental conditions, where many variables can be simultaneously monitored, thereby resulting in comprehensive and precisely analyzed information. Nevertheless, quantifying hunger and satiety under these conditions may be slightly different due to subjects being aware that they are being questioned about them, while measuring them in the real world may not always be possible. The sample size also tends to be low and often limited to a very specific population, such as healthy subjects, only men/women, only students, among others, which makes it difficult to generalize the results. For diet-related effects, cultural, household, and personal variability may also further complicate such generalizations into other populations.

It is therefore important to interpret the data from a given model with caution by being aware of its strengths and limitations; in fact, it may be preferable to gather evidence from multiple sources. The data analyzed in the present work show that in vitro and animal models are more abundant, as compared to human studies; thus, the need for the latter is apparent.

Finally, a note should be made regarding phenolic metabolism. Phenolic compounds are extensively subjected to first pass metabolism in the small intestine and liver (and to a lesser extent in other tissues), where various metabolites are produced, including glucuronidated, sulfated, and methylated forms, among others. The gut microbiota will also metabolize compounds not absorbed by the host or any metabolites that reach the large intestine. Thus, a significant proportion of the originally ingested compound is likely to be converted into various metabolites; these will reach systemic circulation and/or any organs beyond the digestive tract. This finding means that the observed effects regarding an orally ingested phenolic compound, such as those discussed in the present work, may not be entirely due to the compound itself but may result from the action of said metabolites [86,87,88].

Phenolic metabolism has been studied in multiple works; for example, Stalmach et al. [89] showed that approximately 40% of the phenolics present in Concord grape juice reach the large intestine, where they are transformed into 16 distinct metabolites. Additional experiments showed that up to 41 metabolites were detected from the same Concord grape juice, suggesting extensive metabolic pathways [90]. The compounds discussed in the present work have also been shown to go through similar processing after consumption, such as coffee CGA and FA, which are mainly metabolized into sulfates, lactones, and glucuronides [91]. Such processing was shown to be affected by multiple variables, including the role of colonic microorganisms [92] and dose [93].

According to this information, the various bioactivities of phenolics regarding their role on hunger and satiety may be due not just to the parent compounds themselves but to multiple host- and bacterial-derived metabolites. Specifically determining the role of each metabolite is beyond the scope of the present work, but determining the contribution of specific metabolites merits further study. Moreover, many other phenolics are known to be present in everyday food in addition to those discussed here, whose potential interactions among themselves and with the host’s macromolecules are likely to contribute to modulating their effects on hunger and satiety, further adding to the complex interrelation between food and physiological responses to it.

## 8. Conclusions

Phenolic compounds are known to exert multiple health benefits. The literature regarding the four selected phenolic acids discussed in the present work shows that they can modulate hunger and satiety by different mechanisms, including stimulating anorexigenic hormones and their receptors and by inhibiting various enzymatic reactions. Molecular analyses showed that they can form strong interactions that promote said effects, which can be attributed to their chemical structure. Some key structural properties include the presence and distribution of their functional groups, mainly hydroxyl moieties, which favor binding to certain receptors and enzyme active sites. These compounds could be considered for the development of functional foods; however, further studies are needed to conclusively determine additional mechanisms of action by which they could be interacting with different cells, receptors, or enzymes to modulate satiety. In silico analyses could be useful to determine the type of interactions they can form, while in vitro and animal studies are needed to validate their efficacy and safety in living organisms. Future studies may also consider the role of other variables that have an impact on these processes, including phenolic–phenolic or phenolic–protein interactions that may alter a compound’s bioactivities, the role of host- and microbiota-derived metabolites, intracellular mechanisms of action, among others. Such studies will contribute to increasing our understanding of the intricate diet–physiology interrelation.

## Figures and Tables

**Table 1:** Structural characteristics of four selected phenolic acids discussed in the present work and some of their effects on satiety regulation.

Phenolic Acid	Chemical Structure	Log P	Hydrogen Bond Donor Count	Hydrogen Bond Acceptor Count	Reported Effects	References
Chlorogenic acid (CGA)	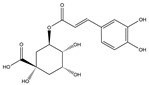	−0.4	6	9	↑ Secretion of GLP-1 ↑ Amylin ↓ MAO-A 5-HT protection ↑ SGLT-1 ↑ Stimulation of L cells	[29,31,32,33,36]
Gallic acid (GA)	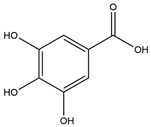	0.7	4	5	↓ Food intake ↓ Octanoyl ghrelin Potential interaction with bitter taste receptors ↓ Body weight ↑ Insulin sensitivity ↓ Diabetic hyperphagia	[40,41,43,44,45]
Ferulic acid (FA)	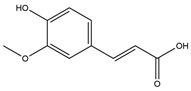	1.5	2	4	May secrete GLP-1 ↓ POMC mRNA ↓ Body weight Modulates intestinal microbiota Inhibition of DPP4 Interactions with GLP-1 ↑ Satiety Modulates GLP-1, leptin, adiponectin mRNA and/or protein Inhibits α-amylase and α -glucosidase	[52,53,55,60,61,62,65]
Protocatechuic acid (PCA)	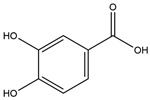	1.1	3	4	↑ PYY, CCK, or GLP1 Modulates intestinal microbiota ↓ Body weight Protects pancreas	[69,70,71,72]

## Data Availability

No new data were created or analyzed in this study. Data sharing is not applicable to this article.

## List of Abbreviations

**Abbreviation**	**Description**
5-HT	5-hydroxytryptamine, serotonin
AgRP	Agouti-related peptide
ARC	arcuate nucleus
AP	avocado paste
CCK1R	CCK type 1 receptor
CHO	Chinese hamster ovary
CGA	chlorogenic acid
CCK	cholecystokinin
CART	cocaine and amphetamine regulated transcript
DPP4	dipeptidyl peptidase 4
FFAR1, also known as GPR-40	fatty acid receptor 1
FA	ferulic acid
FFAR2, also known as GPR-43	free fatty acid receptor 2
FFAR3	free fatty acid receptor 3
GA	gallic acid
GIP	gastric inhibitory polypeptide
GLP-1	glucagon-like peptide-1
GLUT2	glucose transporter 2
GLUT4	glucose transporter 4
GPCRs	G-protein-coupled receptors
hTAS2R	human bitter taste receptors
LEPR	leptin receptor
MC3R	melanocortin receptor 3
MAO-A	monoamine oxidase A
NPY	neuropeptide Y
NF-κB	nuclear factor kappa-light-chain enhancer of activated B cells
PYY	peptide tyrosine tyrosine
Pck1	phosphoenolpyruvate carboxykinase 1
POMC	proopiomelanocortin
PCA	protocatechuic acid
SGLT-1	sodium-glucose cotransporter-1
